# Acute Heat Stress Induces Differential Gene Expressions in the Testes of a Broiler-Type Strain of Taiwan Country Chickens

**DOI:** 10.1371/journal.pone.0125816

**Published:** 2015-05-01

**Authors:** Shih-Han Wang, Chuen-Yu Cheng, Pin-Chi Tang, Chih-Feng Chen, Hsin-Hsin Chen, Yen-Pai Lee, San-Yuan Huang

**Affiliations:** 1 Department of Animal Science, National Chung Hsing University, Taichung, 40227, Taiwan; 2 Agricultural Biotechnology Center, National Chung Hsing University, Taichung, 40227, Taiwan; 3 Center for the Integrative and Evolutionary Galliformes Genomics, iEGG Center, National Chung Hsing University, Taichung, 40227, Taiwan; 4 Department of Veterinary Medicine, National Chung Hsing University, Taichung, 40227, Taiwan; 5 Center of Nanoscience and Nanotechnology, National Chung Hsing University, Taichung, 40227, Taiwan; Wageningen UR Livestock Research, NETHERLANDS

## Abstract

The expression of testicular genes following acute heat stress has been reported in layer-type roosters, but few similar studies have been conducted on broilers. This study investigated the effect of acute heat stress on the gene expression in the testes of a broiler-type strain of Taiwan country chickens. Roosters were subjected to acute heat stress (38°C) for 4 h, and then exposed to 25°C, with testes collected 0, 2, and 6 h after the cessation of heat stress, using non-heat-stressed roosters as controls (n = 3 roosters per group). The body temperature and respiratory rate increased significantly (p<0.05) during the heat stress. The numbers of apoptotic cells increased 2 h after the acute heat stress (79 ± 7 vs. 322 ± 192, control vs. heat stress; p<0.05), which was earlier than the time of increase in layer-type roosters. Based on a chicken 44 K oligo microarray, 163 genes were found to be expressed significantly different in the testes of the heat-stressed chickens from those of the controls, including genes involved in the response to stimulus, protein metabolism, signal transduction, cell adhesion, transcription, and apoptosis. The mRNA expressions of upregulated genes, including *HSP25*, *HSP90AA1*, *HSPA2*, and *LPAR2*, and of downregulated genes, including *CDH5*, *CTNNA3*, *EHF*, *CIRBP*, *SLA*, and *NTF3*, were confirmed through quantitative real-time polymerase chain reaction (qRT-PCR). Moreover, numerous transcripts in the testes exhibited distinct expressions between the heat-stressed broiler-type and layer-type chickens. We concluded that the transcriptional responses of testes to acute heat stress may differ between the broiler-type and layer-type roosters. Whether the differential expression patterns associate with the heat-tolerance in the strains require a further exploration.

## Introduction

A high ambient temperature impairs spermatogenesis and leads to low fertility through a decline of the sperm count, motility, and fertilization rate, as well as an elevation of abnormal cells in domestic animals [1–3]. To understand the adverse effect of high temperature on spermatogenesis, cellular and molecular mechanisms in response to heat stress have been studied [4–8]. Overproduced radical oxygen species that is induced through heat stress has been shown to cause oxidative stress and lead to apoptosis in spermatogenic cells, particularly in spermatocytes [4,7]. Moreover, large amounts of radical oxygen species have been shown to interfere with the integrity of sperm DNA and thereby influence embryo development [7]. A microarray was used to explore the expressions of testicular genes in mouse testes, and the expression levels of genes associated with DNA repair and recombination, protein synthesis, protein folding, and cell cycle reduced after heat stress [5]. Conversely, the expressions of heat-shock protein family genes were augmented against heat stress in mouse testes. Furthermore, a number of genes in testes were shown to be differentially expressed between heat-resistant and heat-sensitive murine strains after heat exposure [6]. Kim et al. [8] argued that multiple mechanisms were involved in heat-shock-induced subfertility or infertility.

In poultry, an environmental temperature of 32 to 35°C has been shown to cause poor fertility through impairing sperm penetration, uterovaginal sperm storage, seminal plasma, and intracellular ion concentrations [9–11]. Because of the subtropical climate of Taiwan, summers are typically characterized by high temperature (up to 38°C) and high humidity [12]. Various local breeds of chicken preserved at National Chung Hsing University exhibit a heat resistance superior to that of foreign breeds [13]. The rectal temperature and blood pH value of Taiwan country chickens (TCCs) were lower than those of broilers when they were subjected to 38°C acute heat stress [14]. A previous study showed that genes participating in response to stress, transport, signal transduction, and metabolism in the testes of L2-strain chickens, a layer-type TCCs, changed after acute heat stress [15]. However, no research has explored the transcriptional responses of genes to acute heat stress in the testes of broilers. In this study, broiler-type chickens of the local breed B-strain TCCs were used to investigate the alteration of global gene expression after exposure to acute heat stress in their testes. The result showed that genes related to heat shock protein family, cell adhesion, transcription, development, and apoptosis were differentially expressed in the testes of heat-stressed B-strain TCCs.

## Materials and Methods

### Experimental animals and ethics statements

Twelve 45-week-old roosters of the B-strain TCC, bred for meat production by National Chung Hsing University [16], were randomly selected and used in this study. The care and use of all animals were complied with the guidelines approved by the Institutional Animal Care and Use Committee (IACUC) of National Chung Hsing University, Taiwan, ROC (IACUC Permit NO. 99–02). Before treatment, roosters were stored in a climate chamber for 2 weeks as an adaptation period under the following conditions: a 14/10-h light/dark photoperiod, 25°C, and 55% relative humidity (RH). Feed and water were provided *ad libitum*.

### Conditions of acute heat stress

The heat treatment was performed according to the procedure of a previous study [15]. After the adaptation period, 9 roosters received acute heat stress (38°C, 55% RH, 4 h) followed by recovery (25°C). Subsequently, the testes of the heat-stressed roosters were collected after 0, 2, and 6 h of recovery and treated as 3 independent groups (H4R0, H4R2, and H4R6, respectively; n = 3 in each group). Controls (n = 3) were maintained at 25°C and 55% RH. Body temperature and respiration rate were recorded during the treatment. For microarray analysis, testis samples were immediately collected and cut into small pieces. The samples were frozen in liquid nitrogen and stored at—80°C until RNA isolation. To prepare tissue sections for apoptosis analysis, the samples were sliced into small pieces, fixed in Bouin’s solution for 24 h, placed into 70% ethanol, and then dehydrated and embedded in paraffin wax.

### Sample collection and preparation

A total of 5 μg of RNA was reverse transcribed using moloney murine leukemia virus reverse transcriptase (Promega, Madison, WI, USA). A total of 0.2 μg of RNA from each sample was used to synthesize second-strand complementary DNA (cDNA), which was amplified using a Quick-Amp Labeling kit (Agilent Technologies, Santa Clara, CA, USA). The cDNA was stored at −80°C until further microarray and quantitative real-time polymerase chain reaction (qRT-PCR) analyses.

### Gene expressions through microarray analysis

RNA samples from 3 animals in each group were separately used for microarray hybridization as a biological triplicate in each treatment. Briefly, cDNA was used as a target for cRNA labeling Cy3-CTP (CyDye, Agilent Technologies) and hybridized with a chicken 44 K oligo microarray (Agilent Technologies) [17]. Using the cDNA as a template enabled labeling the target cRNA. A total of 1.65 μg of Cy3-labled cRNA was fragmented to an average size of approximately 50 to 100 nucleotides through incubation in a fragmentation buffer at 60°C for 30 min. Correspondingly fragmented cRNA was hybridized in a microarray at 65°C for 17 h. Subsequently, the microarray was washed, dried with a nitrogen gun, and scanned using an Agilent microarray scanner (Agilent Technologies) at 535 nm. The scanned images were analyzed using Feature Extraction 10.5.1.1 software (Agilent Technologies) and GeneSpring software (Agilent Technologies); image-analysis and normalization software were used to quantify the signal and background intensity for each feature. Data acquisition was conducted using the following criteria: (1) a false discovery rate of <0.05; (2) p<0.05 for the difference in gene expression; and (3) a distinct signal from the microarray image that was marked with a flag using GeneSpring software. The differentially expressed genes were considered significant if they exhibited a fold-change cut-off that was twice as low or twice as high as that in the control group. The dataset of microarray analysis were submitted to Gene Expression Omnibus in the National Center for Biotechnology Information under an accession number of GSE65279.

### Validation of gene expression through quantitative real-time polymerase chain reaction

The mRNA expressions of 10 differentially expressed genes after heat stress were validated through qRT-PCR. The genes were selected based on that they were commonly differed in both broiler-type (in this study) and layer-type TCCs [15] after acute heat stress, including *HSP25*, *HSPA2* (*HPS70*), *HSP90AA1*, and *CIRBP*. The other genes were selected because of their higher (over 3) fold changes and their relationship to blood-testis barrier, signal transduction, and transcription, including *SLA*, *EHF*, *NTF3*, *CDH5*, *CTNNA3*, and *LPAR2*. Specific oligonucleotide primer pairs were selected from Roche Universal ProbeLibrary for RT-PCR assays (S1 Table). The specificity of each primer pair was validated by performing a RT-PCR reaction using common reference RNA (Stratagene, La Jolla, CA, USA) as a DNA template; the size of the PCR product was checked using a DNA 1000 chip (Agilent Technologies) operated on a Bioanalyzer 2100 (Agilent Technologies). Primer pairs for generating a predicted product size and no other side product were used to conduct the following real-time RT-PCR reaction. Primers for the qRT-PCR and product sizes are listed in S1 Table. The *GAPDH* gene was used as an internal control to normalize the relative expressions of the target genes.

Real-time PCR reactions were performed on the Roche LightCycler Instrument 1.5, using LightCycler FastStart DNA MasterPLUS SYBR Green I kit (Roche Cat. 03 515 885 001, Castle Hill, Australia). Subsequently, 10 μl reactions containing 2 μl of Master Mix, 2 μl of 0.75 μM forward and reverse primers each, and 6 μl of cDNA sample were performed in triplicate. The RT-PCR protocol specified 95°C for 10 min, 40 cycles of 95°C for 10 sec, 60°C for 15 sec, and 72°C for 10 sec. At the end of the program, a melt curve analysis was performed, the data were automatically analyzed by the system, and an amplification plot was generated for each cDNA sample. Based on each of these plots, the LightCycler3 data analysis software automatically calculated a crossing point (CP) value; the turning point corresponded to the first maximum of the second derivative curve, which functioned as the beginning of the exponential amplification. The fold expression or repression of the target gene relative to the internal control gene *GAPDH* in each sample was then calculated using the following formula:

$$2^{-∆∆CP\;}where\;∆Cp\;=\;{Cp}_{target\;gene}-{Cp}_{internal\;control}\;and\;∆∆Cp\;=\;{∆Cp}_{test\;sample}-{∆Cp}_{control\;sample}$$

To ensure consistency with the microarray analysis, the cut-off value for differentially expressed genes was set to ≥ 2-fold change.

### Gene annotation and gene network analysis

The genes with known identities or with homologous sequences and functional definitions were classified according to their cellular components, biological processes, and molecular functions according to annotations in the Gene Ontology (GO) [18] http://www.geneontology.org/]. Differentially expressed genes were mapped to protein interactions of the corresponding genes through functional protein association networks at the String website [19].

### Terminal deoxynucleotidyl transferase dUTP nick end labeling assay

The terminal deoxynucleotidyl transferase dUTP nick end labeling (TUNEL) assay for identifying apoptotic cells was performed using the In Situ Cell Death Detection Kit, Peroxidase (Roche, Mannheim, Germany). Paraffin-embedded testis tissues were sectioned, deparaffinized, and rehydrated with EZ-DeWax. The sections were then subjected to a TUNEL assay according to the standard protocol recommended by the manufacturer. The frequencies of apoptotic cells were calculated in 250 seminiferous tubules per section.

### Statistical analysis

Fold changes in the microarray and qRT-PCR analysis were presented as the arithmetic mean of 3 replicates in each group. For comparison between the control and heat-stress groups, gene expressions with ≥ 2-fold changes (up or down) were considered to be significantly different. The significant difference of the frequency of apoptotic cells was analyzed using the general linear model procedure with Statistical Analysis System software (SAS Institute, Cary, NC, USA).

## Results

### Physical response to acute heat stress in broiler-type TCCs

The body temperature in the B-strain TCCs was higher than that in the controls during 0.5 to 4 h of the acute heat stress (p<0.05; S2 Table), and it recovered to the same level as that of the controls 1 h after the acute heat stress. Panting increased at 1 h of heat exposure (p<0.05) and recovered after 1 h of recovery.

The number of apoptotic testicular cells initially increased in heat-stressed broiler-type TCCs (79 ± 7 vs. 253 ± 103, control vs. H4R0; p<0.1) and showed an elevation after 2 h of recovery unlike those of the controls (322 ± 192 vs. 79 ± 7, H4R2 vs. control; p<0.05). However, the numbers of apoptotic cells did not differ after 6 h of recovery (79 ± 7 vs. 117 ± 60, control vs. H4R6; p>0.05).

### The alteration of global gene expression in the testes of broiler-type TCCs after acute heat stress

To investigate the gene response to acute heat stress in the testes of broiler-type TCCs, microarray analysis was used to explore whole gene expression patterns. The results of the microarray analysis showed that there were 196 probe sets with significant changes in recovery after the acute heat stress (S1 Fig). These differentially expressed probe sets represented 163 unique genes, including 141 known genes and 22 unknown genes with only expressed sequence tags (S3 Table). A total of 77 genes were upregulated but 86 genes were downregulated in the testes of chickens after heat stress (S2 Fig). Moreover, 45, 38, and 19 genes were upregulated, and 71, 16, and 9 genes were downregulated after 0, 2, and 6 h of recovery, respectively.

**Fig 1:**
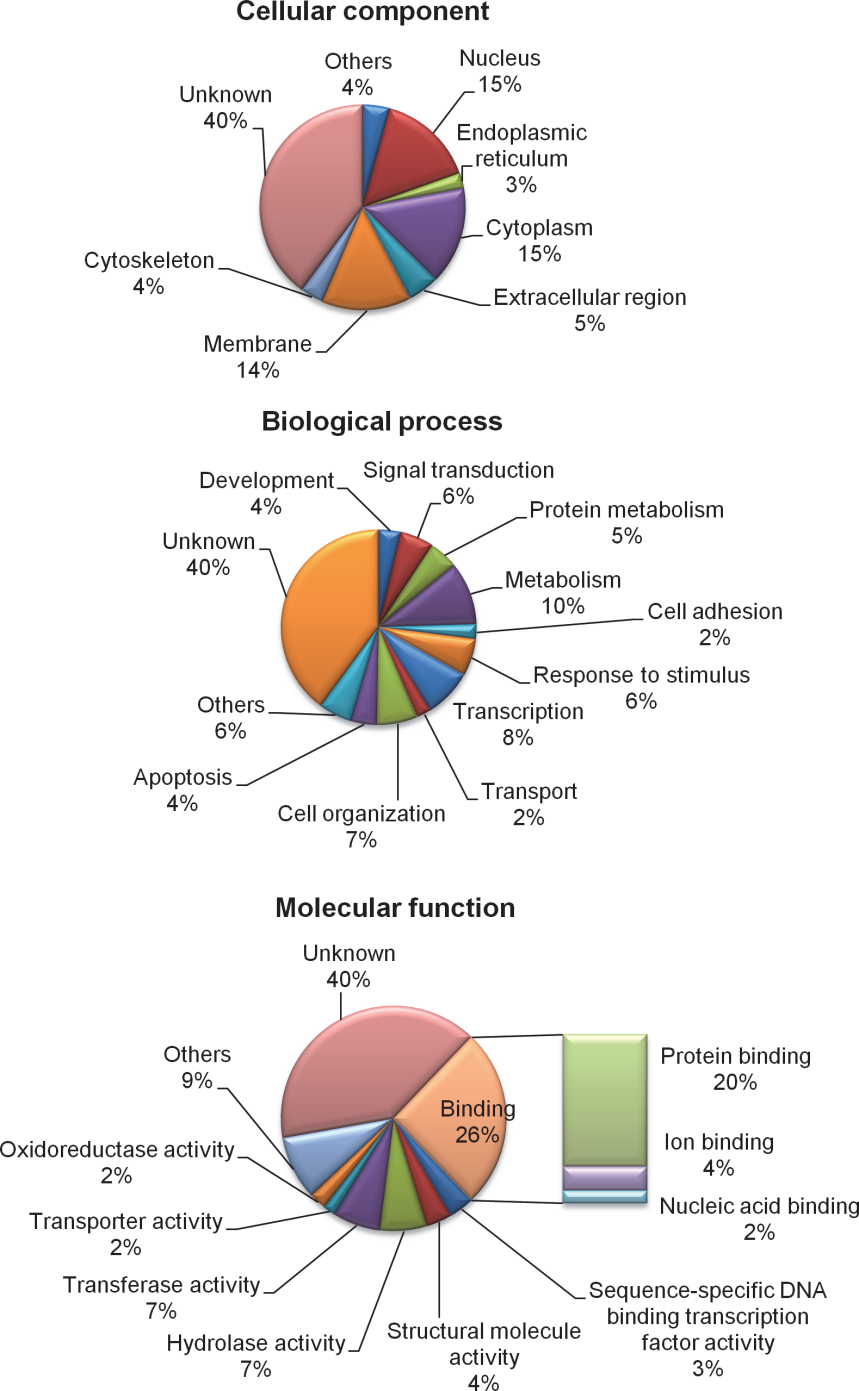
Pie chart showing the classification of differentially expressed genes in the testes of B-strain TCCs after 4 h of acute heat stress and recovery of 0, 2, and 6 h, in terms of cellular components, biological processes, and molecular functions.

**Fig 2:**
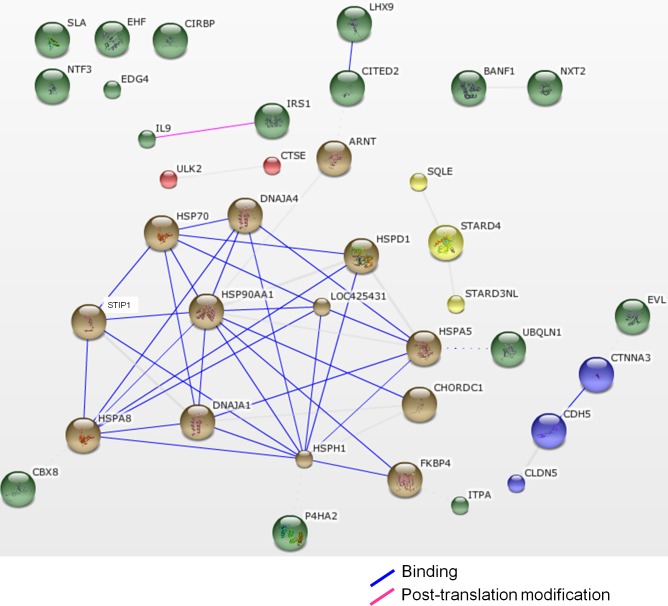
The STRING network of protein interaction in various differentially expressed genes in the testes of heat-stressed B-strain TCCs. The lines between the protein nodes show the actions among the proteins in *Gallus gallus*.

The functions of the differentially expressed genes were defined according to their GO annotations, namely cellular components, biological processes, and molecular functions (Fig 1). The genes were located primarily in the nucleus (15%), cytoplasm (15%), and membrane (14%). Most of the genes participated in the metabolism (10%), transcription (8%), cellular organization (7%), response to stimulus (6%), and signal transduction (6%). The expressions of the genes that were involved in the response to stimulus, such as heat-shock protein 25 (*HSP25*) and heat-shock 70 kDa protein 2 (*HSPA2*), increased after heat stress in chicken testes, while the cold inducible RNA binding protein (*CIRBP*) was downregulated (S3 Table). The results suggested that genes participating in signal transduction were upregulated, including the lysophosphatidic acid receptor 2 (*LPAR2*), or downregulated, including the interleukin 13 receptor, alpha 1 (*IL13RA1*), in the testes of B-strain TCCs after heat stress. Genes involved in transcription also changed in the heat-stressed chicken testes. For example, the Ets homologous factor (*EHF*) was downregulated after heat stress. Genes involved in cell adhesion were also downregulated after heat exposure, including cadherin 5 (*CDH5*), catenin, alpha 3 (*CTNNA3*), claudin 5 (*CLDN5*), and protocadherin-17-like (*LOC100551402*). In the biological process of apoptotic process, the heat-shock 70 kDa protein 5 (*HSPA5*), solute carrier family 5, member 11 (*SLC5A11*), gasdermin A (*GSDMA*), and heat-shock 60 kDa protein 1 (*HSPD1*) increased after heat stress. Regarding molecular functions, the differentially expressed genes were primarily sorted into protein binding (20%), hydrolase activity (7%), transferase activity (7%), and structural molecule activity types (4%; Fig 1). A gene network analysis showed that genes of heat-shock proteins (HSPs) and cochaperones exhibited strong relationships (Fig 2).

### Validation of differentially expressed genes in the testes of broiler-type TCCs after acute heat stress

**Fig 3:**
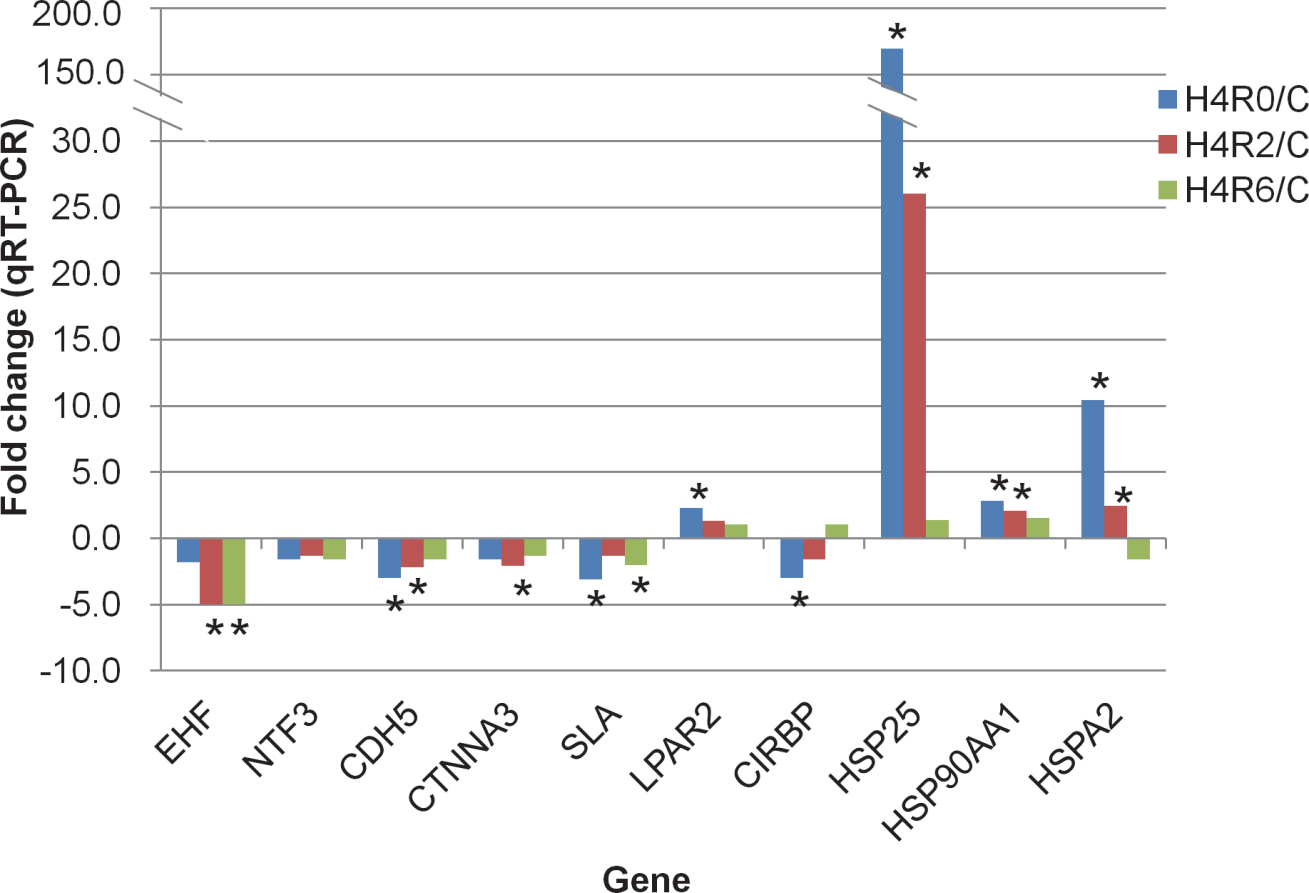
Validation of selected differentially expressed genes through qRT-PCR. The * indicates the expression of a gene with a significant difference (p<0.05) compared with that of the control (C). H4R0, heat-stressed group without recovery; H4R2, heat-stressed group after 2 h of recovery; H4R6, heat-stressed group after 6 h of recovery. *EHF*, Ets homologous factor; *NTF3*, neurotrophin 3; *CDH5*, cadherin 5; *CTNNA3*, catenin alpha 3; *SLA*, Src-like-adaptor; *LPAR2*, lysophosphatidic acid receptor 2; *CIRBP*, cold inducible RNA binding protein; *HSP25*, heat-shock protein 25; *HSP90AA1*, heat-shock protein 90 kDa alpha, class A member 1; *HSPA2*, heat-shock 70 kDa protein 2.

The mRNA expression levels of 10 genes commonly differed in both broiler-type and layer-type TCCs after acute heat stress or with fold changes higher than 3 and relationship to blood-testis barrier, signal transduction, and transcription, namely *EHF*, *NTF3*, *CDH5*, *CTNNA3*, Src-like-adaptor (*SLA*), *LPAR2*, *CIRBP*, *HSP25*, *HSP90AA1*, and *HSPA2*, were determined through qRT-PCR (Fig 3). The mRNA expressions of *HSP25*, *HSP90AA1*, and *HSPA2* increased after 0 and 2 h of recovery in heat-stressed chicken testes. The mRNA level of *LPAR2* was upregulated after 0 h of recovery, after the acute heat stress. In downregulated genes, *CIRBP* exhibited a decline after 0 h and *CTNNA3* changed after 2 h of recovery. The mRNA level of *EHF* decreased after 2 and 6 h of recovery, whereas *CDH5* was downregulated after 0 and 2 h recovery in chicken testes after the acute heat stress. The mRNA expression level of *SLA* decreased after 0 and 6 h of recovery after the heat stress. The mRNA expression of *NTF3* exhibited no alteration in heat-stressed chicken testes. The mRNA expressions of selected genes determined through the qRT-PCR analysis were consistent with the microarray results. Although the mRNA expressions of *NTF3* exhibited no significantly difference, the expression pattern was similar to that identified in the microarray data.

## Discussion

Heat stress impairs sperm motility and fertility in roosters. Few studies have elaborated on the effect of acute heat stress on gene expression in chicken testes. Our previous study on layer-type TCCs showed that 309 genes were differentially expressed in the testes after acute heat stress [15]. The functional network analysis revealed that genes of HSPs and cochaperones (*HSP70*, *HSP90AA1*, *HSP25*, and *DNAJA4*) as well as antiapoptotic *BAG3* and *SERPINB2* were upregulated in heat-stressed chicken testes. The results of this study further showed that broiler-type TCCs exhibited a rapid response to acute heat stress and they recovered immediately after heat stress (S2 Table). The microarray analysis identified 163 genes that were differentially expressed in the testes of broiler-type TCCs after 38°C acute heat stress, most of which changed after 0 h of recovery. The differentially expressed genes participated in transcription, response to stimulus, protein metabolism, cell adhesion, apoptosis, and signal transduction. Moreover, the mRNA levels of the genes were determined through qRT-PCR, and the results were consistent with those of the microarray analysis. In layer-type chickens, genes that expressed differentially were primarily observed after 0 and 2 h of heat stress [15]. We inferred that acute heat stress altered the gene expressions immediately in the testes of broiler-type TCCs.

### Alterations of stress-related genes in the testes of broiler-type TCCs after acute heat stress

The mRNA levels of genes belonging to the heat-shock protein family increased in the testes of broiler-type TCCs after 4 h of heat stress, including *HSP25*, *HSPA2*, *HSP90AA1*, *HSPA8*, *HSPD1*, *HSPH1*, and *HSPA5* (Fig 2 and S3 Table). Heat stress caused an elevation of HSP expression, attenuating the damage [20]. In heat-stressed mice, *HSPA8* expression was shown to increase, protecting the testis from apoptosis and assisting inducible *HSP70* (*HSPA2*) in repairing the stress-induced misfolded proteins [21]. Under acute heat stress, the expression levels of *HSPH1* and *HSP25* in liver, brain, and leg muscle increased to suppress cell death in boilers [22]. A previous study also indicated that mRNA expressions, including those of *HSP25*, *HSPA2*, *HSP90AA1*, *HSPA8*, and *HSPH1*, were upregulated in heat-stressed layer-type roosters [15]. An endoplasmic reticulum stress marker, *HSPA5* [23], was induced in human fibroblasts after 30 min of heat stress [24]. The results of this study suggested that the testes responded to acute heat stress through the induction of HSP gene expressions (especially *HSP25*, *HSPA2*, *HSP90AA1*, *HSPA8*, *HSPH1*, and *HSPA5*) in broiler-type TCCs. Whether the overexpressions of the HSP genes attenuate stress in chickens requires further investigation.

### Genes related to blood–testis barrier and transcription altered in the testes of broiler-type TCCs after acute heat stress

The genes related to the blood–testis barrier, including *CDH5*, *CATNNA3*, *CLDN5*, and *LOC100551402*, decreased at recovery in the testes of heat-stressed broiler-type TCCs (S3 Table). A blood–testis barrier is formed by anchoring Sertoli cells through cell junction proteins to prevent system circulation and allow the transit of preleptotene spermatocytes in the testis [25–28]. Various factors, such as cytokines, signal transducers, hormones, and heat stress, can modulate the expression levels or locations of cell junction proteins and thus regulate the integrity of the blood-testis barrier [27,29–32]. A short-term scrotal heat stress disrupts the integrity of the blood–testis barrier by downregulating the expressions of cell junction proteins, including cadherin, catenin, and claudin in mammals [30,32]. The decreased expressions of genes associated with cell junction in the testes of heat-stressed B-strain TCCs might imply that the integrity of the blood-testis barrier was affected in broiler-type chickens.

Our results showed that the transcripts involved in transcription or signal transduction were changed in the testes of broiler-type TCCs after heat stress (S3 Table). For instance, the mRNA expression level of *EHF* decreased whereas that of *LPAR2* increased in heat-stressed chickens. These genes play essential roles in cell proliferation and differentiation [33–36]. Furthermore, the downregulated *SLA* in the testes of heat-stressed broiler-type TCCs (Fig 3) has been reported to be associated with cell proliferation [37,38]. Previous studies have suggested that *SLA* is a negative regulator of B-cell development and osteoclastogenesis [37,38]. A lack of *SLA* was shown to contribute to the proliferation of osteoclast precursors in mice [38]. The E26 transformation-specific (ETS) gene family is one of the largest families of transcriptional regulators regulating a variety of biological processes in normal cell homeostasis [33]. In the ETS gene family, the *EHF* is also known as epithelial specific *ETS-3* and functions in epithelial cell differentiation and proliferation in mammals [34]. In human prostate epithelial cells, *EHF* can balance cell differentiation and self-renewal [35]. As a member of the G-protein-coupled receptor family, *LPAR* regulates embryogenesis, development, cell survival, immune cell trafficking, and inflammatory reactions [36]. The deletion of *LPAR* results in a loss of sperm production in mice [39]. The downregulated *EHF* and *SLA* but upregulated *LPAR2* in the testes of heat-stressed B-strain TCCs imply that acute heat stress might influence cell proliferation and spermatogenesis of broiler-type chickens.

### Expression difference of genes in the testes of heat-stressed broiler-type and layer-type TCCs in response to acute heat stress

**Fig 4:**
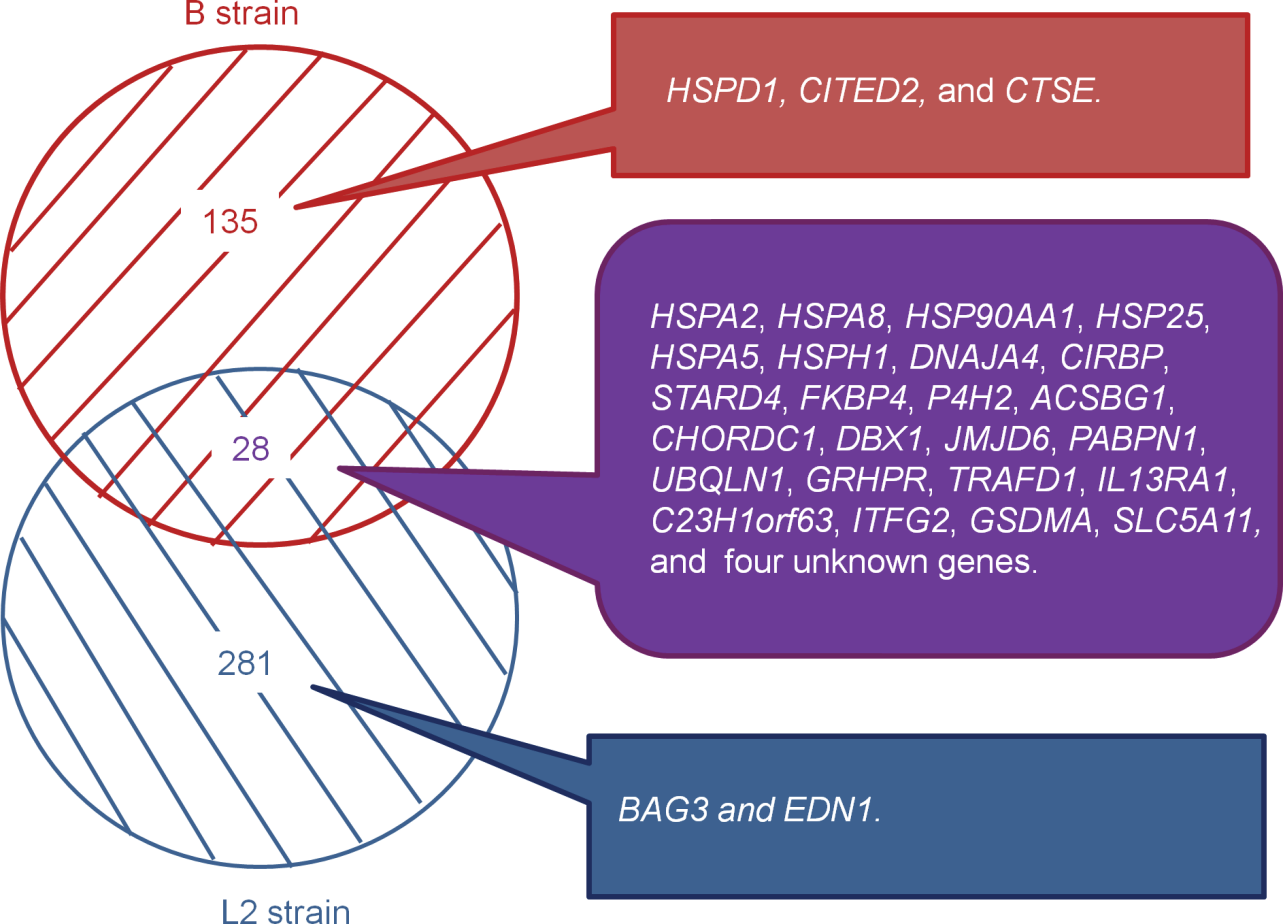
Comparison of differentially expressed genes between B-strain (broiler-type) and L2-strain (layer-type) TCCs after acute heat stress.

Initial heat-induced apoptosis in the testes of broiler-type TCCs was observed after 0 h of recovery after heat exposure. Furthermore, the apoptotic cells in the testes increased significantly after 2 h of recovery in heat-stressed broiler-type TCCs. In the L2 strain of layer-type TCCs, the number of apoptotic testicular cells was significantly higher than that in the controls after only 2 h of recovery [15]. An increase of apoptotic germ cells in heat-resistant mice occurred later than the increase in controls after exposure to heat stress (43°C, 15 min) [6]. The difference between strains might imply that the testicular cells of broiler-type TCCs were more sensitive than those of layer-type chickens when they were subjected to acute heat stress. Further comparison of the differences of testicular gene expressions between broiler-type and layer-type chickens showed that there were 135 genes exhibited substantial differences, specifically in the testes of heat-stressed broiler-type TCCs, whereas 281 genes showed alterations specifically in layer-type TCCs (Fig 4). A total of 28 genes exhibited substantial changes in both types of chickens and most of the genes were HSP genes. Acute heat stress caused various trends of expressions of apoptosis-related genes between the testes of broiler-type and layer-type TCCs. For example, the Cbp/p300-interacting transactivator together with Glu/Asp-rich carboxy-terminal domain 2 (*CITED2*) and *HSPD1* increased but cathepsin E (*CTSE*) decreased in the testes of broiler-type TCCs after acute heat stress. Moreover, in layer-type L2-strain TCCs, antiapoptotic genes were upregulated, including BCL2-associated athanogene 3 (*BAG3*) and endothelin-1 (*EDN1*; Fig 4). The *CTSE*, an aspartic protease, plays a role in suppressing cell growth and metastasis in tumor cells [40]. In human prostate-cancer cell lines, a higher expression of *CTSE* induces growth arrest and apoptosis by catalyzing the proteolytic release of a tumor necrosis factor-related apoptosis-inducing ligand, which induces apoptosis [41]. As a member of the HSP family, *HSPD1* plays multiple roles in cellular processes, including apoptosis, anti-apoptosis, and protein folding [42–44]. The mRNA expression of *HSPD1* is localized in spermatogonia and Sertoli cells in normal monkey testes and elevated to prevent apoptosis after a short-term heat stress [42]. The *BAG3* negatively regulates apoptosis, and it can be induced by heat stress to attenuate apoptosis in chickens [15,45]. The *CITED2* acts as an anti-apoptotic gene, and a lack of *CITED2* increases apoptosis in the anterior region of testes during early gonad development in mice [46]. Furthermore, hypoxic stress-induced apoptosis can be decreased by stimulating the transcription of *CITED2* in fibroblasts [47]. The *EDN1* reveals an anti-apoptotic effect in rat uterine leiomyoma ELT3 cells [48]. Moreover, the expression of *EDN1* augments apoptosis in cancer cells induced by mild hyperthermia [49]. We inferred that the differential gene expression in response to acute heat stress in the testes of broiler-type and layer-type roosters may thus result in various responses to heat stress (e.g., apoptosis). However, whether the different expression patterns associate with the heat-tolerance as well as the cause-effect relationship in the strains require further exploration.

## Conclusion

A total of 163 genes changed in the testes of broiler-type B-strain TCCs after acute heat stress. The differentially expressed genes are primarily involved in the protein metabolism, cell adhesion, transcription, development, and apoptosis; this suggests that heat stress may affect the cell survival, differentiation, and development in the testes of broiler-type chickens. Moreover, the partially different expression patterns of genes in the testes of broiler-type and layer-type chickens may suggest that testicular responses to acute heat stress differ between the two types of chickens. As the functions of genes are reflected in their protein levels and post-translational modifications, the results of this study require further validation at translation levels and a follow-up study is now under investigation.

## Supporting Information

S1 Fig Hierarchical clustering of 196 probe sets differentially expressed at > 2.0-fold changes in the testes of B-strain TCCs receiving acute-heat stress for 4 h and recovery for 0, 2, and 6 h. H4R0, heat stress group after 0 h of recovery; H4R2, heat stress group after 2 h of recovery; H4R6, heat stress group after 6 h of recovery; Control, chickens not subjected to heat stress.
(TIFF)

S2 Fig Venn diagram analysis of upregulated genes (77 genes) and downregulated genes (86 genes) in the testes of B strain TCCs after acute heat stress. The numbers in parentheses represent the amount of differentially expressed genes. H4R0, 0 h of recovery after heat stress; H4R2, 2 h of recovery after heat stress; H4R6, 6 h of recovery after heat stress.
(TIFF)

S1 Table Primers of selected differentially expressed genes in the testes of heat-stressed B strain TCCs used for qRT-PCR. (DOC)

S2 Table Body temperature and respiratory rate of acute heat stressed B strain TCCs during heat stress and recovery period. (DOC)

S3 Table Differentially expressed genes in the testes of B strain TCCs after acute heat stress. (DOC)
