# Supplementary figures and images for: Acute Heat Stress Induces Differential Gene Expressions in the Testes of a Broiler-Type Strain of Taiwan Country Chickens
Source: PLoS One. 2015 May 1;10(5):e0125816. doi: 10.1371/journal.pone.0125816 (PMC4416790; doi:10.1371/journal.pone.0125816)

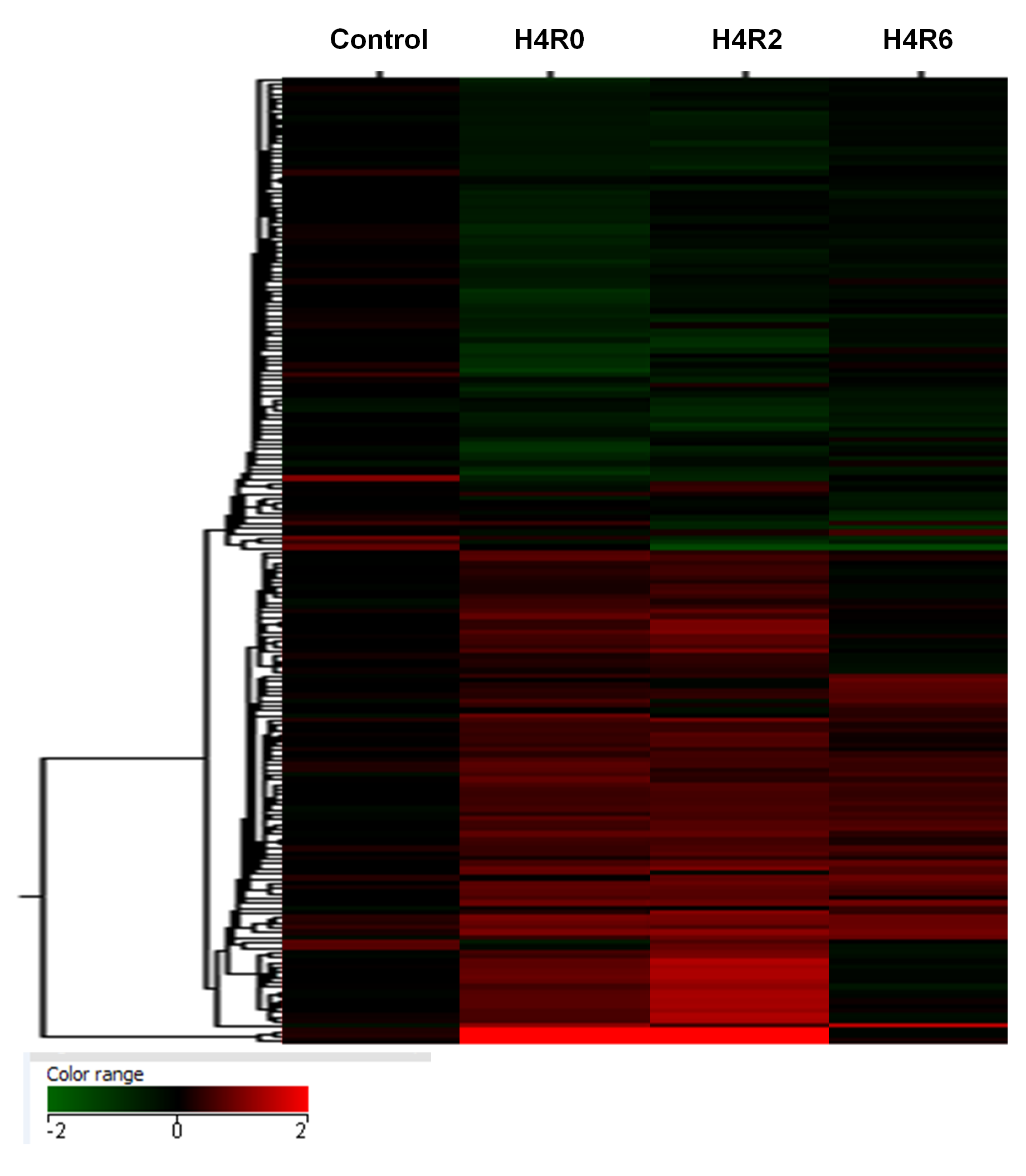

Supplement: S1 Fig — H4R0, heat stress group after 0 h of recovery; H4R2, heat stress group after 2 h of recovery; H4R6, heat stress group after 6 h of recovery; Control, chickens not subjected to heat stress. (TIFF) [file pone.0125816.s001.tiff]

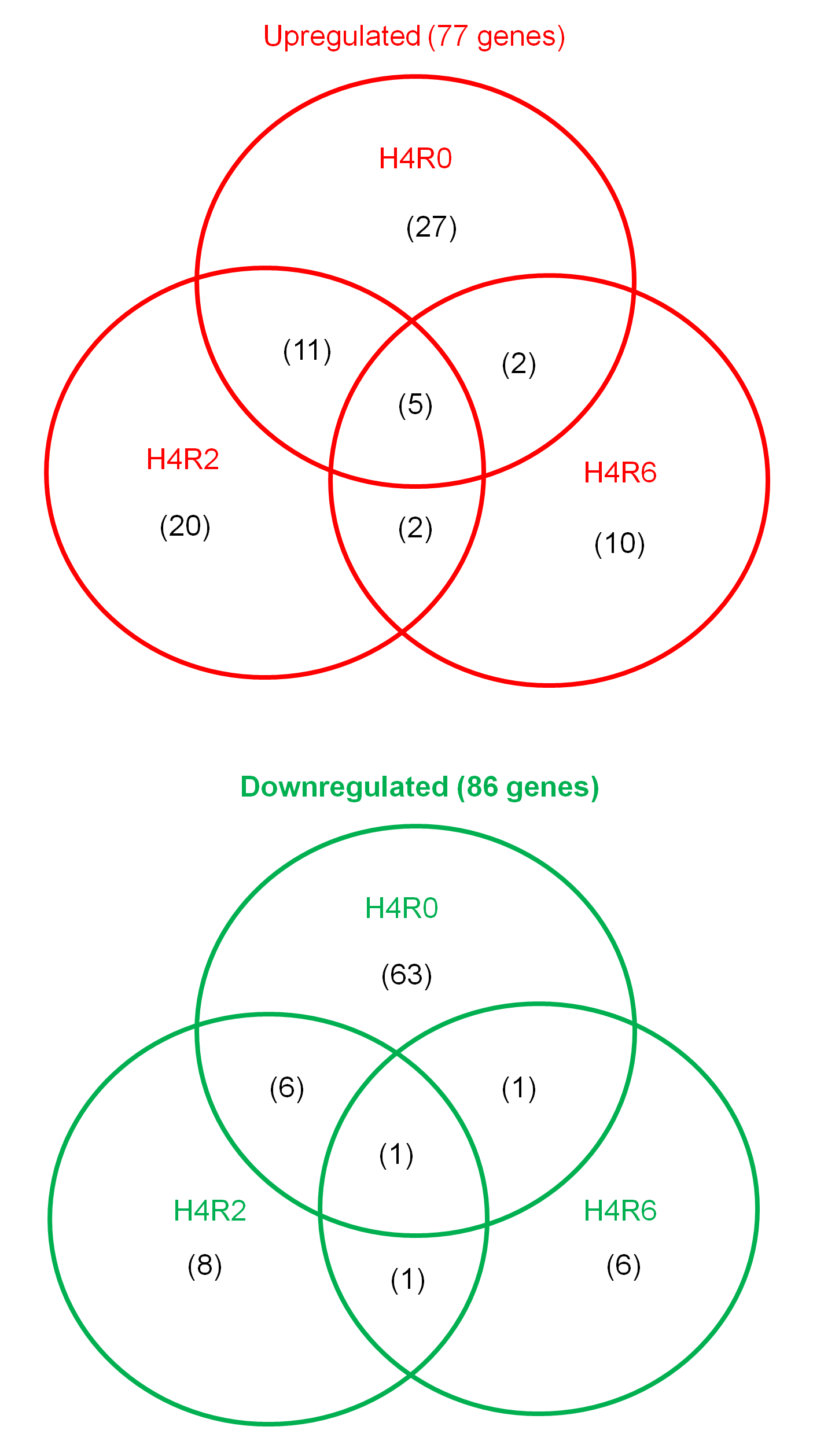

Supplement: S2 Fig — The numbers in parentheses represent the amount of differentially expressed genes. H4R0, 0 h of recovery after heat stress; H4R2, 2 h of recovery after heat stress; H4R6, 6 h of recovery after heat stress. (TIFF) [file pone.0125816.s002.tiff]
