# Supplementary material for: Acute Heat Stress Induces Differential Gene Expressions in the Testes of a Broiler-Type Strain of Taiwan Country Chickens
Source: PLoS One. 2015 May 1;10(5):e0125816. doi: 10.1371/journal.pone.0125816 (PMC4416790; doi:10.1371/journal.pone.0125816)
Supplement: S1 Table — (DOC) [file pone.0125816.s003.doc]

S1 Table. Primers of selected differentially expressed genes in the testes of heat-stressed B strain TCC used for qRT-PCR.

| Probe name | Gene symbol | Description | Primer | Product size (bp) |
| --- | --- | --- | --- | --- |
| A_87_P021775 | EHF | Ets homologous factor | F: TACTCCACCAGCAGCCTTCC  R: CCACTCCCAGACCTGAAACTTTG | 125 |
| A_87_P005314 | NTF3 | Neurotrophin 3, transcript variant 1 | F: AGACTTCCAGCCAGTTATTTCAATG  R: GGTACAGTGGTGGTGGTTCC | 116 |
| A_87_P037572 | CDH5 | Cadherin 5, type 2 | F: ATTTGGGGATGACAGCCTTGG  R: TACTAACGGGTGGAACCTCAATAAC | 108 |
| A_87_P156188 | CTNNA3 | Similar to catenin, alpha 3 | F: TTTGTGCGGTATATTGCTCAGAAG  R: CTTGGAGATACTGGCTTTAAGATGC | 65 |
| A_87_P105283 | SLA | Src-like-adaptor | F: TCAGAGAACAAAGTTTCGTTGGTTG  R: GGCGTGCCTTCAGGAGATG | 143 |
| A_87_P057346 | LPAR2 | Lysophosphatidic acid receptor 2 | F: CAACGCCATCGTTTACTCCT  R: TGGGGGTATATTTGGGGTTC | 98 |
| A_87_P054071 | CIRBP | Cold inducible RNA binding protein | F: GCCTGGGTACAAATTGGAAG  R: GCAGGTTGAACATACAAGCAAG | 72 |
| A_87_P293458 | HSP25 | Heat shock protein 25 | F: CCGTCTTCTGCTGAGAGGAGTG  R: ACCGTTGTTCCGTCCCATCAC | 117 |
| A_87_P085771 | HSP90AA1 | Heat shock protein 90kDa alpha, class A member 1 | F: GGTGTTGGTTCCTACTCTGCTTAC  R: ACTGCTCATCATCATTGTGCTTGG | 76 |
| A_87_P226483 | HSPA2 | Heat shock 70kDa protein 2 | F: CAACCCGATTGTCACAAAACT  R: TTGGCAATGACGTTTTTCCT | 182 |
| Internal control gene | GAPDH | Glyceraldehyde-3-phosphate dehydrogenase | F: CATCACAGCCACACAGAAGA  R: TGACTTTCCCCACAGCCTTA | 122 |
