# Supplementary material for: Acute Heat Stress Induces Differential Gene Expressions in the Testes of a Broiler-Type Strain of Taiwan Country Chickens
Source: PLoS One. 2015 May 1;10(5):e0125816. doi: 10.1371/journal.pone.0125816 (PMC4416790; doi:10.1371/journal.pone.0125816)
Supplement: S2 Table — (DOC) [file pone.0125816.s004.doc]

S2 Table. Body temperature and respiratory rate of acute heat stressed B strain TCCs during heat stress and recovery period.

| Treatment time (h) | Heat treatment (38°C) | | | | | |  | Recovery time (25°C) | | |
| --- | --- | --- | --- | --- | --- | --- | --- | --- | --- | --- |
|  | 0 | 0.5 | 1 | 2 | 3 | 4 |  | 1 | 2 | 6 |
| Body temperature (°C) |  |  |  |  |  |  |  |  |  |  |
| Control | 40.9±0.2 | 40.7±0.1^b^ | 40.8±0.0^b^ | 40.2±0.2^b^ | 40.7±0.2^b^ | 40.8±0.1^b^ |  | 40.7 | 40.1 | 39.9 |
| Heat stress | 41.5±0.1 | 42.3±0.2^a^ | 43.2±0.2^a^ | 43.2±0.3^a^ | 43.3±0.3^a^ | 43.4±0.4^a^ |  | 40.4±0.4 | 40.1±0.2 | 39.7±0.4 |
| Respiratory rate (times/min) |  |  |  |  |  |  |  |  |  |  |
| Control | 28±4 | 25±4 | 27±4^b^ | 28±4^b^ | 21±4 | 23±4^b^ |  | 28 | 28 | 32 |
| Heat stress | 29±3 | 36±4 | 72±15^a^ | 69±15^a^ | 56±9 | 56±7^a^ |  | 31±2 | 29±2 | 32±2 |

Control, non-heat-stressed roosters. Data are presented as mean ± standard error.

^a,b^ Values differ significantly between heat- stressed and control group (p<0.05).
