# Supplementary material for: Acute Heat Stress Induces Differential Gene Expressions in the Testes of a Broiler-Type Strain of Taiwan Country Chickens
Source: PLoS One. 2015 May 1;10(5):e0125816. doi: 10.1371/journal.pone.0125816 (PMC4416790; doi:10.1371/journal.pone.0125816)
Supplement: S3 Table — (DOC) [file pone.0125816.s005.doc]

S3 Table. Differentially expressed genes in the testes of B strain TCCs after acute heat stress.

| Probe name | Fold change | | | Gene symbol | Description* | UniGene ID^#^ |
| --- | --- | --- | --- | --- | --- | --- |
|  | H4R0/C | H4R2/C | H4R6/C |  |  |  |
| Development |  |  |  |  |  |  |
| A_87_P037735 | 2.4 | 3.0 | 1.5 | CITED2 | Gallus gallus Cbp/p300-interacting transactivator, with Glu/Asp-rich carboxy-terminal domain, 2, mRNA [NM_206844] | Gga.2845 |
| A_87_P003926 | 1.9 | 2.4 | 1.0 | RUFY3 | PREDICTED:RUN and FYVE domain containing 3, mRNA [XM_420599] | Gga.15381 |
| A_87_P009110 | 1.7 | 2.3 | 1.2 | CRYBB3 | Gallus gallus crystallin, beta B3, mRNA [NM_205191] | Gga.655 |
| A_87_P005314 | -4.0 | -3.2 | -2.0 | NTF3 | Gallus gallus neurotrophin 3, mRNA [NM_001109762] | Gga.41617 |
| A_87_P130668 | -2.1 | -1.4 | -1.1 | COL9A2 | Collagen, type IX, alpha 2, mRNA [M16715] | Gga.865 |
| A_87_P011183 | 1.3 | -2.2 | -1.4 | SETDB1 | Histone-lysine N-methyltransferase SETDB1-like, mRNA [CR524048] | Gga.499 |
| Signal transduction | |  |  |  |  |  |
| A_87_P057346 | 3.0 | 1.5 | 1.3 | LPAR2 | Gallus gallus Lysophosphatidic acid receptor 2, mRNA [NM_001115081] | Gga.12211 |
| A_87_P007751 | 1.8 | 2.2 | 1.2 | RALGDS | PREDICTED: Gallus gallus ral guanine nucleotide dissociation stimulator, mRNA [XM_425331] | Gga.30795 |
| A_87_P058111 | 1.9 | 2.0 | 1.2 | IGSF1 | Gallus gallus immunoglobulin superfamily, member 1, mRNA [NM_001145226] | Gga.7064 |
| A_87_P305448 | 1.4 | 3.9 | 1.8 | QRFP | PREDICTED: Gallus gallus pyroglutamylated RFamide peptide, mRNA [XM_001235088] | ―――― |
| A_87_P009216 | 1.8 | 2.1 | 1.2 | IRS-1 | Gallus gallus insulin receptor substrate 1, mRNA [NM_001031570] | Gga.32082 |
| A_87_P024122 | -2.2 | -1.8 | 1.1 | C1QTNF2 | C1q and tumor necrosis factor related protein 2, mRNA [BX930999] | Gga.7886 |
| A_87_P070736 | -2.4 | -1.2 | -1.3 | TTI1 | Tel2 interacting protein 1 homolog, mRNA [AJ851644] | Gga.1078 |
| A_87_P016668 | -1.1 | -1.3 | -2.2 | CILP | Cartilage intermediate layer protein, nucleotide pyrophosphohydrolase, mRNA [CR386159] | Gga.15814 |
| Protein metabolism | |  |  |  |  |  |
| A_87_P156813 | 6.7 | 2.3 | -1.3 | HSPH1 | Gallus gallus heat shock 105kDa/110kDa protein 1, mRNA [NM_001159698] | Gga.21366 |
| A_87_P203553 | 4.4 | 2.8 | 1.1 | DNAJA4 | PREDICTED: Gallus gallus DnaJ homolog, subfamily A, member 4, mRNA | Gga.5900 |
| A_87_P322137 | 3.6 | 1.7 | -1.1 | HSPA8 | Gallus gallus heat shock 70kDa protein 8, mRNA [NM_205003] | Gga.4555 |
| A_87_P017936 | 2.2 | -1.0 | 1.5 | Senp3 | SUMO/sentrin specific peptidase 3, mRNA [CR353439] | Gga.14035 |
| A_87_P036470 | 2.1 | 1.5 | -1.0 | FKBP4 | Gallus gallus FK506 binding protein 4, 59kDa, mRNA [NM_001006250] | Gga.9013 |
| A_87_P085771 | 2.3 | 1.8 | 1.4 | HSP90AA1 | Gallus gallus heat shock protein 90kDa alpha, class A member 1, mRNA [NM_001109785] | Gga.6482 |
| A_87_P063571 | -2.4 | -1.3 | -1.4 | CTSE | PREDICTED: Gallus gallus similar to cathepsin E, mRNA [XM_001235023] | Gga.11160 |
| A_87_P009783 | -2.6 | -2.3 | -1.2 | CAPN2 | Gallus gallus calpain 2, (m/II) large subunit, mRNA [NM_205080] | Gga.3677 |
| Metabolic process | |  |  |  |  |  |
| A_87_P235963 | 2.6 | 1.4 | 1.0 | ACSBG1 | Acyl-CoA synthetase bubblegum family member 1, mRNA [BX936010] | Gga.12240 |
| A_87_P059786 | 5.2 | 2.8 | -1.2 | P4HA2 | Gallus gallus prolyl 4-hydroxylase, alpha polypeptide II, mRNA [NM_001006155] | Gga.5799 |
| A_87_P126703 | 2.7 | 3.1 | 1.2 | LEO1 | PREDICTED: RNA polymerase-associated protein LEO1-like, mRNA [CR406690] | Gga.19071 |
| A_87_P024455 | 1.8 | 2.2 | 1.7 | SULT1B1 | Sulfotransferase family, cytosolic, 1B, member 1, mRNA [BX930069] | Gga.8853 |
| A_87_P153768 | 1.4 | 2.0 | 1.8 | SRSF4 | PREDICTED: Gallus gallus similar to SFRS4, mRNA [XM_417747] | Gga.15313 |
| A_87_P143533 | 1.9 | 5.9 | 3.4 | NANP | Gallus gallus N-acetylneuraminic acid phosphatase, mRNA [NM_001031059] | Gga.13530 |
| A_87_P022216 | 1.6 | 2.3 | 2.0 | APRT | PREDICTED: adenine phosphoribosyltransferase-like, mRNA [BX935197] | Gga.6594 |
| A_87_P028831 | 1.9 | 1.3 | 2.3 | ITPA | Inosine triphosphate pyrophosphatase, mRNA [BU370052] | Gga.11897 |
| A_87_P057126 | 1.6 | 1.0 | -2.2 | PLA2G7 | Gallus gallus phospholipase A2, group VII, mRNA [NM_204969] | Gga.42432 |
| A_87_P013828 | -2.1 | -1.4 | -1.0 | KIAA1456 | KIAA1456, mRNA [CR390951] | Gga.17066 |
| A_87_P014366 | -2.2 | -1.1 | 1.0 | HELQ | Helicase, POLQ-like, mRNA [CR390109] | Gga.18271 |
| A_87_P036692 | -2.1 | -1.6 | -1.1 | SQLE | Gallus gallus squalene epoxidase, mRNA [NM_001194927] | Gga.22304 |
| A_87_P070421 | -2.1 | -1.3 | -1.3 | TGM6 | PREDICTED: Gallus gallus transglutaminase 6, mRNA [XM_001234441] | ―――― |
| A_87_P152588 | -2.9 | -1.3 | -1.6 | ZSWIM7 | Zinc finger, SWIM-type containing 7, mRNA [DR420117] | Gga.12656 |
| A_87_P018172 | -3.9 | -2.1 | -2.0 | LOC100859237 | PREDICTED: 2-acylglycerol O-acyltransferase 2-like, partial, mRNA [CR353094] | Gga.14165 |
| A_87_P021585 | -1.3 | -2.2 | -1.4 | TPK1 | Thiamin pyrophosphokinase 1, mRNA [BX950550] | Gga.12854 |
| A_87_P031721 | -1.6 | -2.4 | 1.0 | H6PD | PREDICTED: Gallus gallus similar to glucose 1-dehydrogenase, mRNA [XM_425746] | Gga.44427 |
| Cell adhesion |  |  |  |  |  |  |
| A_87_P010744 | -2.8 | 1.2 | -2.1 | LOC100551402 | PREDICTED: protocadherin-17-like, mRNA [CR733366] | Gga.21900 |
| A_87_P127008 | -2.2 | -1.3 | -1.4 | CLDN5 | Claudin 5, mRNA [CR407183] | Gga.3111 |
| A_87_P037572 | -3.7 | -2.6 | -1.8 | CDH5 | Gallus gallus cadherin 5, type 2, mRNA [NM_204227] | Gga.5171 |
| A_87_P156188 | -1.5 | -3.5 | -1.3 | CTNNA3 | PREDICTED: Gallus gallus similar to catenin, alpha 3, mRNA [XM_001232672] | Gga.19961 |
| Response to stimulus | |  |  |  |  |  |
| A_87_P011124 | 1.0 | -1.2 | -2.3 | DNAJA1 | Gallus gallus DnaJ homolog, subfamily A, member 1, mRNA [NM_001012945] | Gga.5644 |
| A_87_P012034 | 2.3 | 1.7 | 1.6 | STIP1 | PREDICTED: stress-induced-phosphoprotein 1, partial, mRNA [CR407499] | Gga.11348 |
| A_87_P022685 | 2.6 | 2.2 | 2.2 | BANF1 | PREDICTED: barrier-to-autointegration factor-like isoform 1, mRNA [BX934219] | Gga.11427 |
| A_87_P024651 | 3.5 | 1.7 | 1.6 | LOC425431 | PREDICTED: Gallus gallus dnaJ homolog subfamily A member 1-like. [BX929626] | Gga.44537 |
| A_87_P054071 | -3.4 | -1.5 | 1.1 | CIRBP | Gallus gallus cold inducible RNA binding protein , mRNA [NM_001031347] | Gga.4756 |
| A_87_P079786 | 1.4 | 2.3 | 1.4 | RPE65 | Gallus gallus retinal pigment epithelium-specific protein 65kDa, mRNA [NM_204884] | Gga.408 |
| A_87_P119753 | 2.8 | 1.0 | -1.4 | CHORDC1 | Cysteine and histidine-rich domain containing 1, mRNA[CR353453] | Gga.14419 |
| A_87_P163628 | -2.0 | -1.2 | -1.2 | ULK2 | PREDICTED: Gallus gallus unc-51-like kinase 2, mRNA [XM_415858] | Gga.28814 |
| A_87_P226483 | 7.7 | 2.8 | -1.2 | HSPA2 | Gallus gallus heat shock 70kDa protein 2, mRNA [NM_001006685] | Gga.4942 |
| A_87_P293458 | 112.8 | 24.3 | 1.9 | HSP25 | Gallus gallus heat shock protein 25, mRNA [NM_001010842] | Gga.47060 |
| Transcription |  |  |  |  |  |  |
| A_87_P037501 | 2.4 | 2.6 | 2.1 | EBF1 | Gallus gallus early B-cell factor 1, mRNA [NM_204752] | Gga.276 |
| A_87_P002051 | 2.3 | 1.2 | -1.1 | DBX1 | Developing brain homeobox 1, mRNA [XR_026947] | Gga.25865 |
| A_87_P199248 | 2.5 | -1.0 | -1.5 | JMJD6 | Gallus gallus jumonji domain containing 6, mRNA [NM_001030703] | Gga.21114 |
| A_87_P200843 | 2.0 | 1.6 | 1.6 | ARNT | Gallus gallus aryl hydrocarbon receptor nuclear translocator, mRNA [NM_204200] | Gga.8458 |
| A_87_P086501 | 1.7 | 2.3 | 1.5 | CHMP2A | Gallus gallus chromatin modifying protein 2A, mRNA [NM_001080892] | Gga.7026 |
| A_87_P270803 | -1.0 | 1.1 | 2.2 | EAF2 | Gallus gallus ELL associated factor 2, mRNA [NM_001006525] | Gga.19230 |
| A_87_P011151 | -2.2 | -1.4 | -1.1 | LOC418083 | Host cell factor C2, mRNA [CR524095] | Gga.12626 |
| A_87_P012173 | -2.8 | -1.4 | -1.0 | CDX1 | Gallus gallus caudal type homeobox 1, mRNA [NM_204676] | Gga.210 |
| A_87_P013380 | -2.2 | -1.6 | -1.1 | PABPN1 | Poly(A) binding protein, nuclear 1, mRNA [CR391548] | Gga.18812 |
| A_87_P064931 | -2.9 | -1.5 | -1.2 | TRIM29 | PREDICTED: Gallus gallus similar to Tripartite motif-containing 29, mRNA [XM_417892] | Gga.40786 |
| A_87_P216598 | -2.6 | -1.6 | -1.7 | GATA5 | Transcription factor GATA-5 [ENSGALT00000008583] | ―――― |
| A_87_P177498 | -2.5 | -1.8 | -1.6 |  | Response regulator receiver domain protein [TC363964] | ―――― |
| A_87_P102741 | -2.3 | -2.0 | -1.2 | BRD9 | Bromodomain containing 9, mRNA [XM_418893] | Gga.28402 |
| A_87_P021775 | -1.2 | -8.7 | -2.3 | EHF | Ets homologous factor, mRNA [BX936167] | Gga.12036 |
| Transport |  |  |  |  |  |  |
| A_87_P319492 | 2.0 | 1.7 | 1.7 | LOC425295 | PREDICTED: Gallus gallus similar to Chloride channel protein 2, mRNA [XM_423073] | Gga.26075 |
| A_87_P012759 | -2.0 | -1.1 | -1.1 | AQP12 | Gallus gallus aquaporin 12, mRNA [NM_001109679] | Gga.19694 |
| A_87_P202498 | -2.4 | -1.4 | -1.1 | STARD4 | Gallus gallus StAR-related lipid transfer domain containing 4, mRNA [NM_001079742] | Gga.15316 |
| A_87_P025158 | -1.9 | -1.4 | -2.0 | KCNG4 | Potassium voltage-gated channel, subfamily G, member 4, mRNA [XM_425129] | Gga.6454 |
| Cell organisation | |  |  |  |  |  |
| A_87_P131458 | 2.3 | 1.2 | -1.0 | Tuba1b | Gallus gallus brain alpha-tubulin mRNA, 3'-untranslated region, mRNA [U85490] | Gga.35012 |
| A_87_P237073 | 2.2 | 1.7 | 1.4 | CORO1C | Gallus gallus coronin, actin binding protein, 1C, mRNA [NM_001039265] | Gga.8826 |
| A_87_P036127 | 1.7 | 2.6 | 1.8 | EVL | Gallus gallus Enah/Vasp-like, mRNA [NM_001006487] | Gga.15399 |
| A_87_P080961 | 1.7 | 2.1 | 1.5 | SLIT1 | Slit homolog 1, mRNA [AF364044] | Gga.2156 |
| A_87_P085551 | 1.6 | 1.4 | 3.4 | CCDC88C | PREDICTED: Gallus gallus coiled-coil domain containing 88C, mRNA [XM_421320] | Gga.12289 |
| A_87_P011999 | -2.1 | -1.6 | -1.3 | LHX9 | LIM homeobox 9, mRNA [CR407573] | Gga.2348 |
| A_87_P016435 | -2.4 | -1.3 | -1.2 | SACS | Spastic ataxia of Charlevoix-Saguenay, mRNA [CR386505] | Gga.12671 |
| A_87_P016682 | -2.1 | -1.6 | -1.0 | KANK4 | PREDICTED: KN motif and ankyrin repeat domain-containing protein 4, mRNA [CR386137] | Gga.42922 |
| A_87_P023686 | -2.5 | -1.5 | -1.2 | CLRN1 | Clarin 1, mRNA [BX932032] | Gga.12216 |
| A_87_P035917 | -2.1 | -1.2 | -1.3 | CBX8 | PREDICTED: chromobox protein homolog 8, mRNA [AJ722285] | Gga.46203 |
| A_87_P063326 | -2.3 | -1.2 | -1.0 | NAV1 | neuron navigator 1 [ENSGALT00000000493] | ―――― |
| Apoptotic process | |  |  |  |  |  |
| A_87_P019393 | 2.2 | 1.4 | -1.3 | UBQLN1 | Ubiquilin 1, mRNA [XM_425028] | Gga.5208 |
| A_87_P035946 | 2.6 | 1.9 | 2.7 | GSDMA | Gallus gallus gasdermin A, mRNA [NM_001031361] | Gga.43182 |
| A_87_P269578 | 2.2 | 1.3 | -1.3 | HSPD1 | Gallus gallus heat shock 60kDa protein 1, mRNA [NM_001012916] | Gga.39003 |
| A_87_P071621 | 2.9 | 3.1 | 1.2 | SLC5A11 | Solute carrier family 5, member 11 [TC321992] | ―――― |
| A_87_P037649 | -2.3 | -1.6 | -1.2 | FABP1 | Gallus gallus fatty acid binding protein 1, liver, mRNA [NM_204192] | Gga.3688 |
| A_87_P195928 | 2.5 | 2.1 | -1.1 | HSPA5 | Gallus gallus heat shock 70kDa protein 5, mRNA [NM_205491] | Gga.4219 |
| A_87_P082956 | -2.3 | -1.3 | -1.2 | LOC424261 | Gallus gallusTransforming growth factor, beta receptor II-like, mRNA [BX933582] | Gga.12594 |
| Others |  |  |  |  |  |  |
| A_87_P020440 | 1.5 | 1.3 | 2.1 | DIXDC1 | PREDICTED: Gallus gallus DIX domain containing 1, mRNA [XM_417934] | Gga.14668 |
| A_87_P023862 | -1.6 | -2.2 | -1.3 | GRHPR | Glyoxylate reductase/hydroxypyruvate reductase, mRNA [BX931577] | Gga.35242 |
| A_87_P024051 | -2.6 | -1.6 | 1.0 | IL9 | Gallus gallus interleukin 9, mRNA [NM_001037825] | Gga.11039 |
| A_87_P037110 | -2.1 | -1.4 | 1.0 | TRAFD1 | Gallus gallus TRAF-type zinc finger domain containing 1, mRNA [NM_001006191] | Gga.22366 |
| A_87_P052816 | -2.8 | -1.8 | -1.3 | TNFSF8 | Gallus gallus tumor necrosis factor (ligand) superfamily, member 8, mRNA [NM_204409] | Gga.5181 |
| A_87_P058496 | -2.3 | -1.4 | -1.7 | NES | Gallus gallus nestin, mRNA [NM_205033] | Gga.1899 |
| A_87_P118093 | 2.1 | 1.8 | 1.2 | LOC484869 | Bromodomain containing 4, mRNA [BX950312] | Gga.12482 |
| A_87_P024973 | 1.7 | 2.3 | 2.3 | SPEG | Uncharacterized protein SPEG [TC371505] | ―――― |
| A_87_P084421 | 2.1 | 1.4 | 1.5 | ABTB2 | Ankyrin repeat and BTB domain containing 2 [ENSGALT00000018959] | ―――― |
| Unknown |  |  |  |  |  |  |
| A_87_P060861 | 3.1 | 3.9 | 2.3 | LOC430658 | PREDICTED: Gallus gallus similar to keratin, mRNA [XM_428211] | Gga.6957 |
| A_87_P175963 | 2.6 | 3.4 | 2.1 | LOC420543 | PREDICTED: Gallus gallus hypothetical LOC420543, mRNA [XM_429770] | ―――― |
| A_87_P305098 | 2.0 | 2.2 | 1.5 | LOC770291 | PREDICTED: Gallus gallus hypothetical protein LOC770291, mRNA [XM_001233552] | ―――― |
| A_87_P010936 | 2.3 | 2.1 | -1.2 | LOC100859812 | PREDICTED: uncharacterized protein LOC100859812, mRNA [CR524385] | Gga.21010 |
| A_87_P117374 | 2.7 | 1.1 | -2.1 | LOC419592 | PREDICTED: uncharacterized protein LOC419592, mRNA [BX935301] | Gga.1137 |
| A_87_P160498 | 2.2 | 1.6 | 1.2 | ITFG2 | PREDICTED: Gallus gallus similar to Integrin alpha FG-GAP repeat containing 2, mRNA [XM_416486] | Gga.25638 |
| A_87_P302943 | 2.4 | 1.4 | 1.2 | LOC428578 | PREDICTED: Gallus gallus hypothetical LOC428578, mRNA [XM_426135] | ―――― |
| A_87_P304373 | 2.1 | 1.1 | 1.5 | LOC770218 | PREDICTED: Gallus gallus hypothetical protein LOC770218, mRNA [XM_001233552] | ―――― |
| A_87_P121088 | 2.0 | 1.2 | -1.1 | LOC100859882 | Uncharacterized LOC100859882, mRNA [CR385569] | Gga.43023 |
| A_87_P067211 | -1.2 | 2.2 | -1.4 | MXRA7 | Matrix-remodelling associated 7, mRNA [CR388525] | Gga.37067 |
| A_87_P079446 | 1.0 | 2.4 | 1.8 | LOC772245 | Uncharacterized LOC772245, mRNA [DR417014] | Gga.39669 |
| A_87_P188863 | 1.8 | 2.1 | 1.6 | LOC431044 | PREDICTED: Gallus gallus similar to prolin rich protein, mRNA [XM_428596] | ―――― |
| A_87_P302552 | 1.9 | 2.2 | 1.2 | LOC769961 | PREDICTED: Gallus gallus hypothetical protein LOC769961, mRNA [XM_001233275] | ―――― |
| A_87_P303118 | 1.9 | 2.2 | 1.3 | LOC772585 | PREDICTED: Gallus gallus hypothetical protein LOC772585, mRNA [XM_001235698] | ―――― |
| A_87_P111593 | 1.6 | -1.1 | 2.2 | LOC418995 | Gallus gallus finished cDNA, clone ChEST864b4, mRNA [CR389619] | Gga.45584 |
| A_87_P177238 | 1.7 | 1.4 | 2.3 | AXDND1 | PREDICTED: Gallus gallus axonemal dynein light chain domain containing 1, mRNA [XM_422266] | |
| A_87_P015118 | 1.3 | 1.3 | 2.0 | LOC100858019 | Uncharacterized LOC100858019, mRNA [CR388957] | Gga.17883 |
| A_87_P024174 | -1.3 | -1.1 | 2.1 | LOC100857277 | Uncharacterized LOC100857277, mRNA [BX930810] | Gga.5977 |
| A_87_P304273 | -2.2 | -2.0 | -2.1 | LOC771924 | PREDICTED: Gallus gallus hypothetical protein LOC771924, mRNA [XM_001235133] | ―――― |
| A_87_P008872 | -2.5 | -1.8 | 1.0 | RSFR | Gallus gallus leukocyte ribonuclease A-2, mRNA [NM_001007942] | Gga.34358 |
| A_87_P013636 | -2.4 | -1.8 | -1.6 | LOC426456 | Uncharacterized LOC426456, mRNA [CR391215] | Gga.18678 |
| A_87_P095946 | -2.1 | -1.4 | -1.4 | LOC422725 | PREDICTED: Gallus gallus uncharacterized LOC422725, mRNA [XM_429982] | ―――― |
| A_87_P105283 | -3.0 | -1.2 | -1.8 | SLA | Src-like-adaptor, mRNA [AJ721107] | Gga.8508 |
| A_87_P302008 | -2.4 | -1.0 | 1.4 | LOC768368 | PREDICTED: Gallus gallus hypothetical protein LOC768368, mRNA [XM_001231248] | ―――― |
| A_87_P302018 | -2.5 | -1.3 | -1.2 | LOC768452 | PREDICTED: Gallus gallus hypothetical protein LOC768452, mRNA [XM_001231248] | ―――― |
| A_87_P302063 | -2.2 | -1.4 | -1.2 | LOC770934 | PREDICTED: Gallus gallus hypothetical protein LOC770934, mRNA [XM_001234246] | ―――― |
| A_87_P080686 | -2.3 | -1.3 | 1.1 | TMEM72 | transmembrane protein 72 [ENSGALT00000010131] | ―――― |
| A_87_P127463 | -2.4 | -1.6 | -1.4 | ZC3H11A | Zinc finger CCCH-type containing 11A | Gga.7680 |
| A_87_P311442 | 1.8 | -2.0 | -1.5 | NAT10 | Gallus gallus N-acetyltransferase 10, mRNA [NM_001012940] | Gga.15786 |
| A_87_P228673 | -1.7 | -2.2 | -1.7 | STARD3NL | STARD3 N-terminal like, mRNA [BX935588] | Gga.11436 |
| A_87_P307543 | -1.4 | -1.1 | -2.0 | LOC777503 | PREDICTED: Gallus gallus hypothetical protein LOC777503, mRNA [XM_001236948] | ―――― |
| A_87_P014884 | 2.2 | 2.0 | 1.6 |  | Gallus gallus finished cDNA, clone ChEST729o23. [CR389344] | Gga.18004 |
| A_87_P106286 | 3.8 | 4.6 | 2.9 |  | Uncharacterized protein [ENSGALT00000013638] | ―――― |
| A_87_P100566 | 3.4 | 1.6 | 6.3 |  | Uncharacterized protein [ENSGALT00000036734] | ―――― |
| A_87_P099521 | 2.2 | 1.9 | 1.4 |  | Uncharacterized protein [ENSGALT00000041368] | ―――― |
| A_87_P094931 | 2.2 | 1.7 | -1.1 |  | Uncharacterized protein [ENSGALT00000038636] | ―――― |
| A_87_P015758 | 2.1 | 1.4 | 1.1 |  | Gallus gallus finished cDNA, clone ChEST503m7. [CR387527] | Gga.14574 |
| A_87_P015165 | 1.9 | 2.3 | 1.5 |  | Gallus gallus finished cDNA, clone ChEST84i16. [CR388884] | Gga.16866 |
| A_87_P064441 | 1.9 | 2.3 | 1.5 |  | Uncharacterized protein [ENSGALT00000040833] | ―――― |
| A_87_P012476 | 1.4 | -1.0 | 2.2 |  | Gallus gallus finished cDNA, clone ChEST939c12. [CR406863] | Gga.19804 |
| A_87_P113953 | 1.1 | -1.1 | 2.3 |  | Gallus gallus finished cDNA, clone ChEST966e11. [BX930936] | Gga.10718 |
| A_87_P067471 | -2.8 | -2.3 | -1.5 |  | Uncharacterized protein [ENSGALT00000034608] | ―――― |
| A_87_P010751 | -2.0 | -1.2 | -1.2 |  | Gallus gallus finished cDNA, clone ChEST926d24. [CR733340] | Gga.19787 |
| A_87_P010780 | -2.3 | -1.1 | -1.5 |  | Gallus gallus finished cDNA, clone ChEST537d15. [CR733252] | Gga.21308 |
| A_87_P010910 | -2.2 | -1.5 | -1.5 |  | Gallus gallus finished cDNA, clone ChEST716k3. [CR524415] | Gga.21022 |
| A_87_P011070 | -2.0 | -1.6 | -1.3 |  | Gallus gallus finished cDNA, clone ChEST904o3. [CR524190] | Gga.36286 |
| A_87_P011174 | -2.4 | -1.2 | -1.2 |  | Gallus gallus finished cDNA, clone ChEST1024b4. [CR524060] | Gga.20924 |
| A_87_P011610 | -3.1 | -1.2 | -1.6 |  | Gallus gallus finished cDNA, clone ChEST763d5. [CR523439] | Gga.20717 |
| A_87_P011846 | -2.0 | -1.3 | -1.3 |  | Gallus gallus finished cDNA, clone ChEST568a8. [CR523088] | Gga.36333 |
| A_87_P013112 | -2.6 | -1.4 | -1.1 |  | Gallus gallus finished cDNA, clone ChEST198l5. [CR405918] | Gga.19545 |
| A_87_P013325 | -3.6 | -1.4 | -1.1 |  | Gallus gallus finished cDNA, clone ChEST893o3. [CR391627] | Gga.36060 |
| A_87_P014462 | -2.3 | -1.7 | -1.7 |  | Gallus gallus finished cDNA, clone ChEST997o7. [CR389964] | Gga.17431 |
| A_87_P015014 | -2.0 | -1.2 | -1.1 |  | Gallus gallus finished cDNA, clone ChEST836j23. [CR389129] | Gga.17942 |
| A_87_P016592 | -2.3 | -1.8 | -1.0 |  | Gallus gallus finished cDNA, clone ChEST54c1. [CR386273] | Gga.35386 |
| A_87_P017322 | -2.4 | -1.2 | -1.1 |  | Gallus gallus finished cDNA, clone ChEST405o1. [CR385140] | Gga.15294 |
| A_87_P017435 | -2.3 | -1.5 | -1.5 |  | Gallus gallus finished cDNA, clone ChEST293f8. [CR354267] | Gga.13814 |
| A_87_P017587 | -2.0 | -1.3 | -1.2 |  | Gallus gallus finished cDNA, clone ChEST458f19. [CR354034] | Gga.13888 |
| A_87_P018255 | -2.1 | -1.5 | -1.3 |  | Gallus gallus finished cDNA, clone ChEST539h20. [CR352965] | Gga.38194 |
| A_87_P041786 | -3.1 | -1.5 | -1.3 |  | Uncharacterized protein [ENSGALT00000016206] | ―――― |
| A_87_P070266 | -2.4 | 1.9 | -1.0 |  | Uncharacterized protein [ENSGALT00000040069] | ―――― |
| A_87_P079726 | -2.0 | 1.0 | -1.2 |  | Uncharacterized protein [ENSGALT00000037957] | ―――― |
| A_87_P102101 | -2.6 | -1.3 | -1.1 |  | Uncharacterized protein [ENSGALT00000018740] | ―――― |
| A_87_P111803 | -2.6 | -1.1 | 1.1 |  | Uncharacterized protein [ENSGALT00000030046] | ―――― |
| A_87_P120518 | -2.2 | -1.4 | -1.1 |  | Gallus gallus finished cDNA, clone ChEST295m20. [CR354252] | Gga.13821 |
| A_87_P016003 | -1.7 | -2.0 | -1.9 |  | Gallus gallus finished cDNA, clone ChEST272f21. [CR387169] | Gga.14688 |

*Description including gene identity, GenBank accession number (ex: NM_001012940), EST identification (ex: ChEST997o7), TIGR ID (ex: TC363964), or Ensembl ID (ex: ENSGALT00000040069).

^#^ Gene without UniGene ID is presented “――――”.

Blank in Gene symbol means without official gene name.
